# GRAMMAR-Lambda Delivers Efficient Understanding of the Genetic Basis for Head Size in Catfish

**DOI:** 10.3390/biology14010063

**Published:** 2025-01-13

**Authors:** Yunfeng Zhao, Jin Gao, Hong Feng, Li Jiang

**Affiliations:** 1 Hainan Fisheries Innovation Research Institute, Chinese Academy of Fishery Sciences, Sanya 572024, China; 2 Key Laboratory of Aquatic Genomics, Ministry of Agriculture and Rural Affairs, Beijing Key Laboratory of Fishery Biotechnology, Chinese Academy of Fishery Sciences, Beijing 100141, China; 3 Hainan Academy of Ocean and Fisheries Sciences, Haikou 571126, China; gaojin427@126.com; 4 Department of Paediatrics and Adolescent Medicine, The University of Hong Kong, Hong Kong SAR, China; fenghong@hku.hk

**Keywords:** linear mixed model, EMMAX, *Ictalurus punctatus*, morphology, genome-wide association study

## Abstract

The shape of the skull plays a crucial role in the evolution and adaptation of species to their environments. In the case of aquaculture fish, the size of the head is also an important economic trait, as it is linked to fillet yield and ornamental value. This study applies our GRAMMAR-Lambda method to perform a genome-wide association study analysis on loci related to head size in catfish. Compared with traditional GWAS methods, the GRAMMAR-Lambda method offers higher computational efficiency, statistical power, and stability, especially in complex population structures. This research identifies many candidate genes closely related to cranial morphology in terms of head length, width, and depth in catfish, including *bmpr1bb*, *fgfrl1b*, *nipbl*, *foxp2*, and *pax5*, etc. Based on the results of gene–gene interaction analysis, we speculate that there may be frequent genetic interactions between chromosome 19 and chromosome 29 in bone development. Additionally, many candidate genes, gene families, and mechanisms (such as SOCE mechanisms) affecting skeletal development and morphology have been identified. These findings contribute to our understanding of the genetic architecture of head size and will support marker-assisted breeding in aquaculture, also reflecting the potential application of the GRAMMAR-Lambda method in genetic studies of complex traits.

## Simple Summary

Understanding the genetic factors that influence physical traits in aquaculture species can help improve their commercial value. In this study, we explored the genetic basis of head size in catfish, an important trait linked to yield and aesthetic value. Using an advanced genome-wide association study analysis method called GRAMMAR-Lambda, we identified several key genes and genetic interactions associated with head morphology, which influence cranial structure and bone development. Additionally, we discovered potential interactions between specific chromosomes that may play a role in skeletal growth. These findings not only enhance our understanding of the genetic mechanisms underlying head size in catfish but also provide valuable insights for selective breeding programs in aquaculture, ultimately benefiting the industry and consumers.

## 1. Introduction

Skull morphology and body conformation are critical factors in biological evolution and environmental adaptation. These traits, influenced by genes and their interactions with the environment, play essential roles in survival strategies and behaviors. To better understand the genomic regions controlling head shape and size, previous studies have explored the genetics of skull morphology in various species, including fish, frogs, dogs, rodents, and humans [1,2,3,4,5,6]. In aquaculture species, head size is particularly important as it directly affects fillet yield. Smaller head size and uniform body conformation increase the fillet ratio, which is highly valued in the industry. Therefore, identifying genetic markers associated with head shape and size is essential for breeding strategies and aquaculture improvements. Genome-wide association studies (GWASs) have become a common approach for identifying genetic variations associated with complex traits in aquaculture species and have been extensively applied to analyze traits such as growth [7], fillet yield [8], fillet composition [9], sex determination [10], and head size [2,11]. GWASs on head morphology and size in fish species have included catfish [11], common carp (*Cyprinus carpio*) [2], and bighead carp (*Hypophthalmichthys nobilis*) [12].

In a previous GWAS on catfish head size [11], hybrid catfish were used as experimental fish. These fish were hybrids of female channel catfish (*Ictalurus punctatus*) and male blue catfish (*Ictalurus furcatus*). Typically, channel catfish have larger heads than blue catfish, and the F1 hybrids exhibit heterosis, with faster growth, greater disease resistance, and higher fillet yield [13,14]. This hybrid system provides an ideal model for studying head shape and size. The study used the EMMAX linear mixed model to analyze the data. However, as an approximation method, EMMAX can produce inaccurate results when closely related individuals are included in the data and when related single nucleotide polymorphisms (SNPs) have a significant contribution to phenotypic variance. In that study, no significant quantitative trait nucleotides (QTNs) were identified using EMMAX, and the family-based association test for quantitative traits (QFAM) used showed obvious false positives, as indicated by QQ plots and genomic control values. We acknowledge and appreciate the foundational work of the original researchers, even though they were constrained by the analytical tools available at the time.

To overcome these limitations and obtain more accurate results, we adopted an efficient and robust genome-wide association study approach developed by our team, GRAMMAR-Lambda [15]. Compared with traditional GWAS methods, GRAMMAR-Lambda has several advantages, including simplified computational complexity, elimination of the need to estimate variance components or genomic heritability, increased statistical power through a joint association analysis, and greater stability and statistical reliability under varied QTN numbers and complex population structures. Using GRAMMAR-Lambda, this study reanalyzed the previous catfish data and identified significant QTNs and candidate genes directly impacting head morphology and skull development.

In addition to a single-trait GWAS, multi-trait GWAS strategies that consider the combined information of multiple traits can reveal more genetic information in the genetic analysis of multi-phenotype traits [16,17]. It is well known that many genes can control the variation of multiple traits, a phenomenon known as pleiotropy [18]. Previous studies have shown that jointly analyzing multiple correlated traits can increase the statistical power for identifying pleiotropic genes and those that affect only a single correlated trait [19]. Given the correlations among head length, head width, and head depth, this study also conducted a multi-trait GWAS analysis using the GMAT software (version 1.01) [20].

GWASs have mapped numerous genetic variants associated with various traits. However, these variants explain only a small portion of the phenotypic variance for most complex traits. Gene–gene interactions, also known as epistasis, are considered a potential explanation for “missing heritability” [21,22]. Statistically, two-locus interactions are defined as cases where the combined genotype value of two loci deviates from the sum of single-locus genotype values [23]. The effect of a single gene may be zero or too weak to detect, yet it may significantly affect phenotypic variation when combined with other genes [24]. Given that hybrid catfish are used in this study, the REMMAX function in the GMAT was also employed to conduct epistasis analysis, further exploring candidate genes associated with head shape and size [25].

## 2. Materials and Methods

### 2.1. Sample Profile and Data Preparation

The study population from the previous research consisted of nine families of 1-year-old catfish, generated from the backcross of male F1 hybrid catfish (produced by mating female channel catfish with male blue catfish) and female channel catfish [11]. The offspring were raised together. A total of 556 fish were randomly selected, and their blood samples were collected. The head size, including head length, head width, and head depth, was measured as the trait of interest. After DNA extraction, genotyping was performed using the Affymetrix Axiom catfish 250 K SNP array [26]. According to the previous study, no samples were excluded due to low quality or call rates below 95%. In addition to filtering out SNPs with genotyping errors based on Mendelian laws, a minor allele frequency (MAF) below 5%, or call rates below 95%, we also identified SNPs that were mostly or entirely heterozygous, which were not filtered out by a MAF < 5% but did result in a variance of 0 or close to 0. To filter these SNPs out, we used a variance greater than 0.09 (var > 0.09), which resulted in 184,507 SNPs being retained. To eliminate the effect of body weight and between-family phenotypic stratification, the phenotypic data in the backcross population were adjusted with the cubic root of body weight by simple linear regression within each family. The residuals were used as adjusted phenotypes to carry out the GWAS. The dataset used in this study was downloaded from the public database figshare (https://figshare.com/s/7ad1c3f2a3d3cca9fcbe, accessed on 1 December 2024). For more information on the data, refer to reference [11].

### 2.2. Statistical Analysis

In this study, the capabilities of five methods were compared: GRAMMAR-Lambda (Grl) [15], GRAMMAR-Lambda-joint (Grl-joint), EMMAX, QFAM (conducted in PLINK) [11], and GEMMA, with EMMAX and QFAM being the analysis methods used in the original article. Prior to the association analysis, missing values in the phenotype were filled with the mean of the remaining phenotype values. The EMMAX, QFAM, and GEMMA methods were performed with family information as covariates, while GRAMMAR-Lambda removed the fixed effect of family. The proportion of phenotypic variance explained by one quantitative trait locus (QTL) was calculated as R^2^ = $\sigma_{QTL}^{2}/\sigma_{P}^{2}$ ($\sigma_{QTL}^{2}$ is the effect variance of the QTL and $\sigma_{P}^{2}$ is the phenotype variance). The significance level for genome-wide significance was set at 2.71 × 10^−7^ [−log_10_(*p*-value) = 6.57] based on a Bonferroni correction of 0.05 divided by the number of SNPs (184,507). As for the QFAM (conducted in PLINK) method used in previous research, the threshold of −log_10_(*p*-value) for suggestive association remained set at 5 to demonstrate our replication of the results of the original study. After identifying the QTN, the ±150 kb regions surrounding the QTN were examined for candidate genes.

### 2.3. GRAMMAR-Lambda Implementation

The model used in the implementation is defined as follows:

y=za+g+e

where **y** is a vector of the adjusted phenotype; a is a scalar representing the genetic effect of the tested SNPs on the phenotype; **z** is a vector of indicator variables for SNP genotypes, typically coded as 0, 1, or 2 for the three genotypes AA, AB, and BB; **g** is a vector of polygenic effects excluding the tested SNP, with **g**: *N*(**0**, **K**$\sigma_{g}^{2}$), where $\sigma_{g}^{2}$ is the polygenic variance and **K** is the GRM among individuals; and **e** is the residual error, distributed as **e**: *N*(**0**, **I**$\sigma_{e}^{2}$), where $\sigma_{e}^{2}$ is the residual variance and **I** is the identity matrix.

GRAMMAR-Lambda, like GRAMMAR [20], uses the null model to estimate GBVs:

y=g+e

and then infers the association between the resulting phenotypic residuals and the tested SNP using a simple regression model:

$$y-\hat{g}=za+e$$

The test statistic is formulated as *U*, which is then subjected to a standard normal distribution; in what follows, we transform the normal *U* to Chi-squared statistic $\chi^{2}=U^{2}$ in order to evaluate genomic control.

As in the first stage of GRAMMAR, we estimate the GBVs instead of the polygenic effects. It is known that GBVs include not only polygenic effects but also a portion of QTN effects. Removing partial QTN effects from phenotypic residuals can lead to deflated genome-wide test statistics in the second stage, resulting in significant false-negative errors. This issue is particularly pronounced when the GEBVs are accurately estimated for populations with complex structures. Consequently, the test statistic *U* will be subject to:

*U: N*(0, *λ*)

instead of following the standard normal distribution, where λ represents the variance deflation factor (known as genomic control). Using the frequentist outlier test, we can effectively manage the deflation of the $\chi^{2}$ test statistics by applying:

$$\chi_{c}^{2}=\frac{\chi^{2}}{\lambda }$$

where *λ* is estimated as the mean or median of $\chi^{2}$ obtained from the regression model. $\chi_{c}^{2}$ follows a Chi-squared distribution with 1 degree of freedom.

For the residual $y-\hat{g}$ obtained from the GBLUP, we also conduct a joint association analysis of multiple QTN candidates to increase the statistical power for detecting QTNs. We use backward regression to optimize the following multiple linear model:

$$y-\hat{g}=Z_{c}a_{c}+e$$

where $Z_{c}a_{c}$ is the regression term for the QTN candidates. Based on the Bonferroni-corrected significance level, the genetic effects are selected stepwise, and the corresponding QTNs are identified according to the corrected statistics.

For more information on the GRAMMAR-Lambda method, please refer to our team’s published article [15]. The GRAMMAR-Lambda software (version 1.0) can be obtained for free at the following URL: https://github.com/RunKingProgram/GRAMMAR-Lambda (accessed on 1 December 2024).

### 2.4. Multi-Trait GWAS and Epistasis Analysis Using GMAT Software

We conducted a multi-trait GWAS using the GMAT software package [20], employing the default parameters set by the software. The Genomic Multivariate Analysis Tool (GMAT) is an efficient method for genome-wide association studies (GWASs) of multiple correlated traits. The GMAT can handle incomplete multivariate data, reducing time complexity to *O*(*n*) per SNP, while maintaining high statistical power by allowing the inclusion of individuals with missing phenotypic records. Compared with GEMMA, the GMAT significantly improves computational efficiency, achieving similar detection power but at much faster speeds, making it suitable for large datasets with millions of SNPs.

Additionally, we performed a genome-wide epistatic association analysis using the REMMAX function [25,27] in the GMAT software, utilizing its default parameters. The REMMAX (Rapid Epistatic Mixed-Model Association eXpedited) method is designed for genome-wide epistatic association studies, efficiently analyzing interactions between SNP pairs while controlling for multiple polygenic effects. It improves on previous methods by simultaneously handling additive-by-additive, dominance-by-dominance, and additive-by-dominance epistasis, as well as accounting for intrasubject fluctuations. We identified candidate gene loci in additive-by-additive epistasis and additive-by-dominance epistasis, while no candidate gene loci were found in dominance-by-dominance epistasis or any other analyses.

## 3. Results

### 3.1. Genome-Wide Association Study Analysis

**Figure 1:**
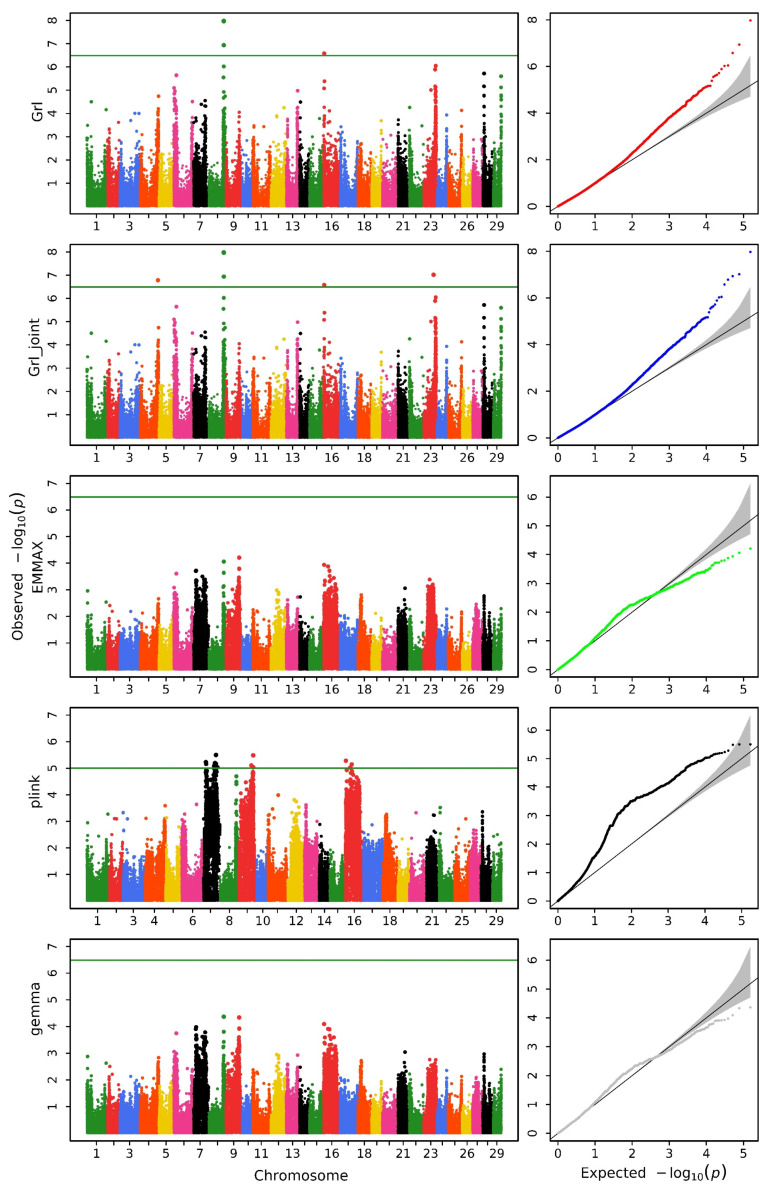
Manhattan (**left**) and QQ (**right**) plots for head length obtained using GRAMMAR-Lambda (Grl), GRAMMAR-Lambda-joint (Grl-joint), EMMAX, QFAM (conducted in PLINK), and GEMMA. The horizontal line in the Manhattan plots indicates the genome-wide significance threshold based on the Bonferroni correction.

**Figure 2:**
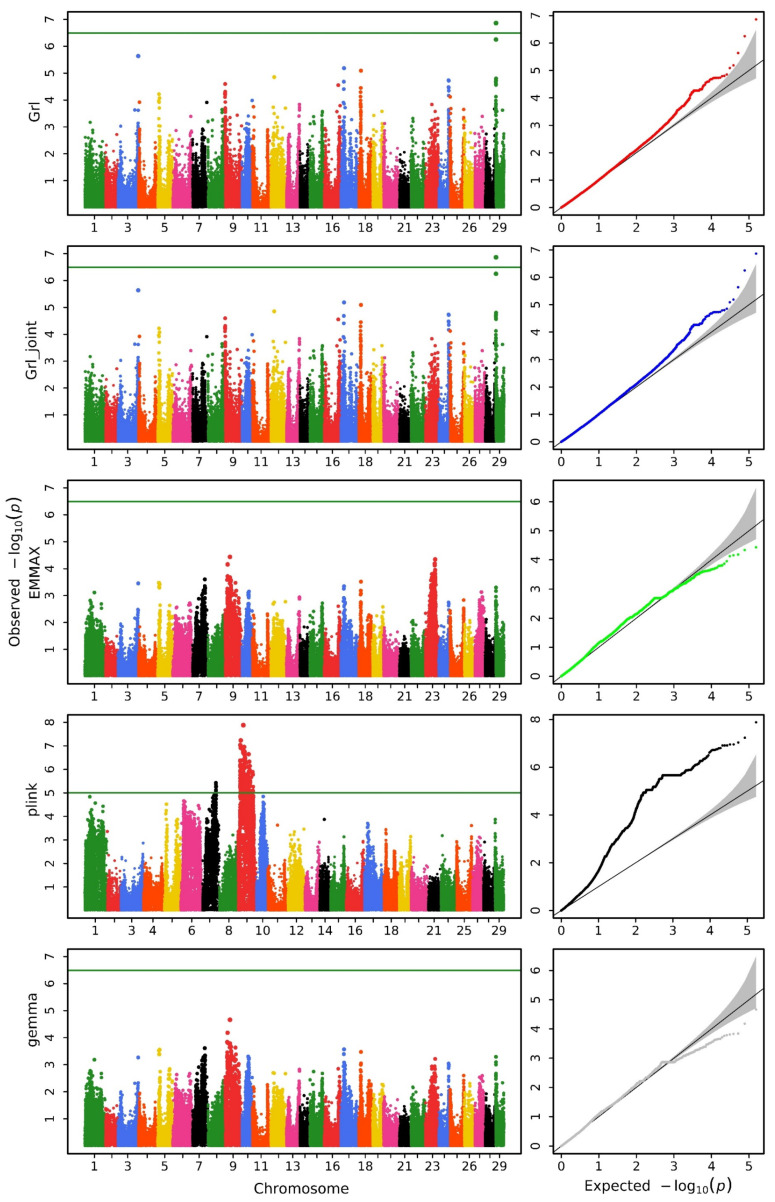
Manhattan (**left**) and QQ (**right**) plots for head width obtained using GRAMMAR-Lambda (Grl), GRAMMAR-Lambda-joint (Grl-joint), EMMAX, QFAM (conducted in PLINK), and GEMMA. The horizontal line in the Manhattan plots indicates the genome-wide significance threshold based on the Bonferroni correction.

**Figure 3:**
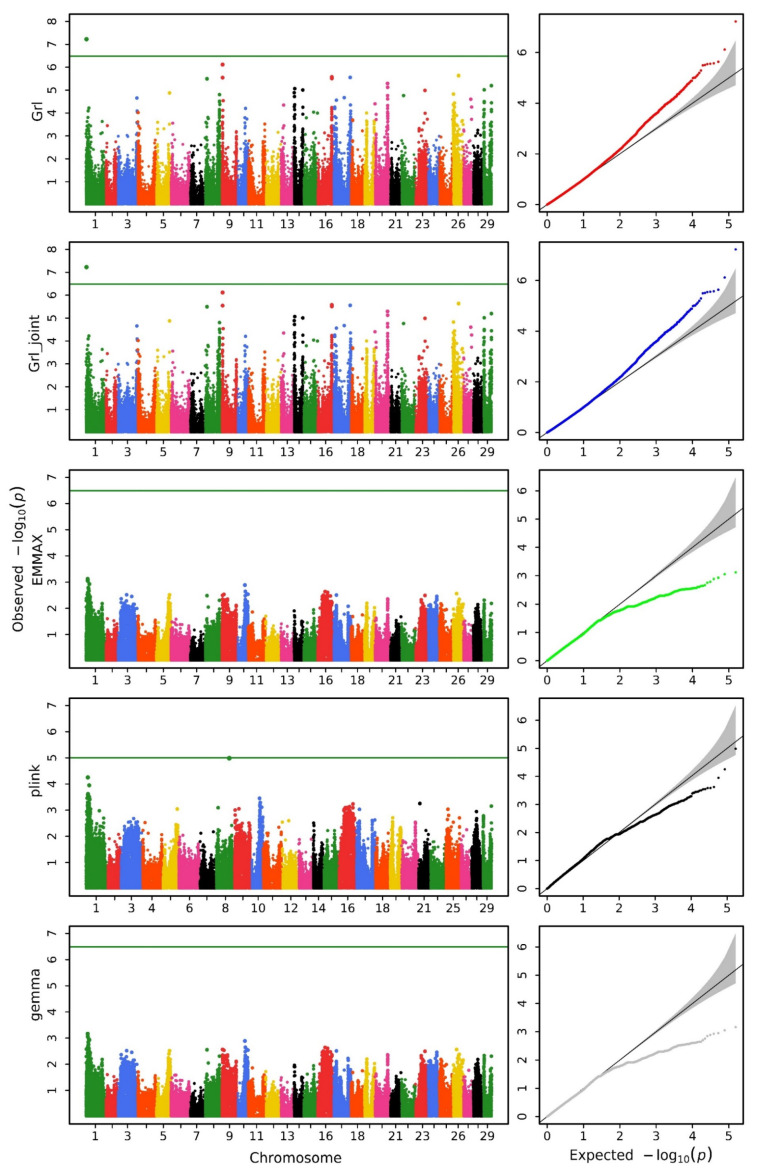
Manhattan (**left**) and QQ (**right**) plots for head depth obtained using GRAMMAR-Lambda (Grl), GRAMMAR-Lambda-joint (Grl-joint), EMMAX, QFAM (conducted in PLINK), and GEMMA. The horizontal line in the Manhattan plots indicates the genome-wide significance threshold based on the Bonferroni correction.

We utilized various methods to conduct a GWAS analysis on the data. Figure 1, Figure 2 and Figure 3 display the Manhattan and QQ plots for the traits of head length, head width, and head depth, using the GRAMMAR-Lambda, GRAMMAR-Lambda-joint, EMMAX, QFAM (conducted in PLINK), and GEMMA methods. The corresponding genomic controls (means of genome-wide Chi-squared statistics) are shown in Table 1. The QQ plots and genomic control values suggest that both the GRAMMAR-Lambda and the GRAMMAR-Lambda-joint methods have desirable statistical properties, whereas the other methods either deflate or inflate test statistics. When referencing the Manhattan plots on the left sides of Figure 1, Figure 2 and Figure 3, the GRAMMAR-Lambda and the GRAMMAR-Lambda-joint methods both identified 5, 1, and 1 QTNs for head length, head width, and head depth, while the EMMAX and GEMMA methods failed to detect any QTNs (Table 2). For the complete table, including genes with unknown functions, please see Table S3.

Our EMMAX analysis result here is consistent with the EMMAX analysis result in the previous research article [11]. The original article conducted analyses using both the EMMAX and the QFAM (conducted in PLINK) methods and overlaid the Manhattan plot results of the two methods in a single figure (Figures S1 and S2) [11]. Upon careful observation of this figure, it can be noted that the EMMAX method in the original article did not identify any QTNs surpassing the Bonferroni correction threshold. The QTNs obtained in the original article primarily originated from the analysis results of the QFAM (conducted in PLINK) method. The authors of the original article also mentioned that they would describe the characters of identified regions according to the results generated from QFAM [11]. In order to compare the analysis results of the original article, we reproduced the analysis of the data using the QFAM (conducted in PLINK) method and identified nearly all QTNs mentioned in the original article. The Manhattan plots were also almost entirely consistent (Figure 1, Figure 2 and Figure 3). However, we observed significant false positives in the QQ plots for the head length and head width traits, while the head depth trait exhibited clear false negatives, which should account for the failure of the original article to identify QTNs in the head depth trait.

The data used in this study were obtained from the Affymetrix Axiom SNP array. The original article listed candidate genes within the ±1 Mb region surrounding the most significant SNPs (Tables S1 and S2) [11]. Here, we used a relatively stricter and closer range to the QTN, listing the associated genes within a ±150 kb range upstream and downstream of the QTN. We identified many genes directly related to bone development, not just those indirectly related through pathways. Among these candidate genes that affect skeletal development, some are even genes that directly influence the development and morphology of the skull (Table 2). For example, the bone morphogenetic protein receptor type IBb (*bmpr1bb*) gene is a member of the Bone Morphogenetic Protein (BMP) receptor family, which is consistent with the head morphological traits we study. The fibroblast growth factor receptor-like 1b (*fgfrl1b*) gene, the deletion of which in zebrafish (*Danio rerio*) results in craniofacial malformations, including the inhibition of cartilage formation in the branchial arches [28]. The *nipbl* gene encodes a protein associated with Cordelmann syndrome, manifesting as craniofacial abnormalities and finger deformities [29]. In the zebrafish pectoral fin (forelimb), the knockdown of *nipbl* gene expression led to size reductions and patterning defects [30]. The *ptx3* gene is significantly involved in the pathogenesis of age-related bone diseases, such as osteoporosis, in both mice and humans [31]. It plays a role in bone metabolism, bone homeostasis, and repair, and it is associated with bone turnover and fractures [32,33]. The *ptx3* gene is also involved in osteoblast differentiation, bone mineralization, and calcification processes [34,35]. The *rpz* gene mutations result in the zebrafish mutant Rapunzel, which exhibits heterozygous defects in bone development, leading to skeletal overgrowth [36]. This finding suggests that Rapunzel 4 (*rpz4*) and Rapunzel 5 (*rpz5*) may be involved in bone development. N-acetylneuraminic acid synthase—a (*nansa*) gene knockdown in zebrafish—leads to abnormal skeletal development in embryos, similar to the phenotype observed in patients [37]. The Iroquois homeobox gene family, including *irx2* and *irx4*, promotes osteoblastogenesis and inhibits adipogenesis [38,39], thereby influencing skeletal development. Additionally, *irx4* is closely associated with bone density and osteoporosis [40]. Studies on zebrafish and mice have shown that *Irx* genes are essential for the development of facial cartilage and the pharyngeal arches [41]. Abnormal functioning of the peptidase D (*pepd*) gene leads to Phosphodiesterase D deficiency (PD). Patients with PD exhibit skeletal deformities such as an abnormal smallness of the head and abnormalities of the nose [42]. These genes that we identified, which directly influence skull morphology or skeletal morphology, were not recognized in the original article. In addition to genes that affect bone morphology, there are candidate genes involved in regulating certain physiological processes of osteoblasts or osteoclasts. For example, tetraspanin 5 (*tspan5*) has been shown to be expressed in osteoclast precursors and is upregulated by RANKL, indicating its role as a positive regulator of osteoclast maturation [43].

In addition to the genes affecting skeletal development and morphology mentioned above, we also identified the SOCE-associated regulatory factor (*saraf*) gene and the stromal interaction molecule 2 (*stim2*) gene from the SOCE mechanism (Store-Operated Calcium Entry). The SOCE mechanism directly influences the function of skeletal cells and the development of skeletal tissue by regulating intracellular calcium levels. The *saraf* gene is a transmembrane protein located in the endoplasmic reticulum (ER) that plays a crucial role in regulating SOCE [44]. It prevents calcium overfilling of the cell by promoting SOCE inactivation. The *saraf* gene interacts with *stim1* and *stim2*, which are essential components of the SOCE machinery, and modulates their function [45]. This interaction is vital for the regulation of the calcium release–activated calcium (CRAC) channel, which is involved in calcium signaling in various cell types, including skeletal muscle cells. In our GWAS results, we found both the *saraf* gene and the stromal interaction molecule 2a (*stim2a*). Research into the role of the *saraf* gene in aquaculture species is still scarce. However, a 2023 study by Xie et al. investigated the function of the *saraf* (SOCE-associated regulatory factor) gene in fish species and highlighted its association with calcium ion transport and mitochondrial dynamics. The *saraf* gene was found to be significantly upregulated in response to low temperature stress in both *C. bouderius* and *P. hypophthalmus*. This suggests that the *saraf* gene is involved in maintaining calcium ion homeostasis and modulating dynamic changes in mitochondrial fusion and fission, particularly under cold stress conditions [46].

Additionally, we identified multiple genes from families involved in skeletal development, including the *SLC* and *Rab* gene families. The *SLC* gene family (Solute Carrier gene family) plays a key role in solute transport, such as ions, nutrients, and small molecules, which are crucial in various physiological processes. According to previous studies, these genes are associated with skeletal development. For example, solute carrier family 6 member 3 (*slc6a3*) has been implicated in canine hip dysplasia, with associated SNPs found in genes that contain *slc6a3* [47]. Solute carrier family 7 member 11 (*slc7a11*) controls cellular reactive oxygen species levels and redirects mitochondrial metabolites, which are necessary for osteoclast function [48]. Additionally, *slc7a11* is involved in the osteogenic differentiation of mesenchymal stem cells and bone formation [49]. Inhibiting *slc7a11* has been shown to enhance osteogenic differentiation of mesenchymal stem cells and promote bone formation [50]. Solute carrier family 24 member 2 (*slc24a2*), known as the sodium–calcium–potassium exchanger [51], is associated with calcium phosphate metabolism, which is crucial for the osteogenic differentiation of stem cells and the formation of bone tissue [52]. The solute carrier family 34 member 2 (*slc34a2*) gene is known to play a crucial role in phosphate homeostasis and bone mineralization, indicating its potential relevance to skeletal development [53,54]. The *Rab* gene family plays a crucial role in cellular membrane transport, having a particularly significant impact on the regulation of osteoclast function [55]. Our GWAS results identified three genes from the *Rab* family: RAB11a, member RAS oncogene family, like (*rab11al*), RAB25, member RAS oncogene family b (*rab25b*), and RAB33A, member RAS oncogene family (*rab33a*). This is consistent with previous research findings. Simultaneously identifying multiple genes from a gene family that influence skeletal development, to some extent, demonstrates the effectiveness of our method. This also reflects the concentrated distribution and coordinated expression characteristics of gene loci affecting the same trait.

### 3.2. Multi-Trait Genome-Wide Association Study Analysis

Through the multi-trait GWAS using the GMAT software, we identified a QTN (AX-85401435) at position 22,832,965 on chromosome 8, with a *p*-value of 2.628 × 10^−12^, surpassing the significance threshold after Bonferroni correction (Table 3). Around this QTN locus (±150 kb), we identified several candidate genes, including *rab33a*, solute carrier family 7 member 5 (*slc7a5*), and ubiquitin-like modifier activating enzyme 2 (*uba2*). The *Rab* gene family has a significant impact on the regulation of osteoclast function [55]. The *slc7a5* gene, which encodes the L-type amino acid transporter 1 (LAT1), is associated with skeletal development. Studies have shown that *slc7a5* plays a role in nutrient signaling in skeletal muscle [56], osteogenic differentiation of bone marrow mesenchymal stem cells [57], and bone homeostasis through the mTORC1 pathway [58]. Additionally, research has indicated that *slc7a5* knockout mice developed osteoporosis [57]. Furthermore, the inactivation of *slc7a5* has been linked to scoliosis in mice [59]. These findings collectively suggest that *slc7a5* is involved in skeletal development and maintenance. The *uba2* is also related to skeletal development. Variants in *uba2* have been associated with a syndrome characterized by limb deformities such as ectrodactyly [60]. For the complete table, including genes with unknown functions, please see Table S4.

### 3.3. Genome-Wide Epistatic Association Analysis

**Figure 4:**
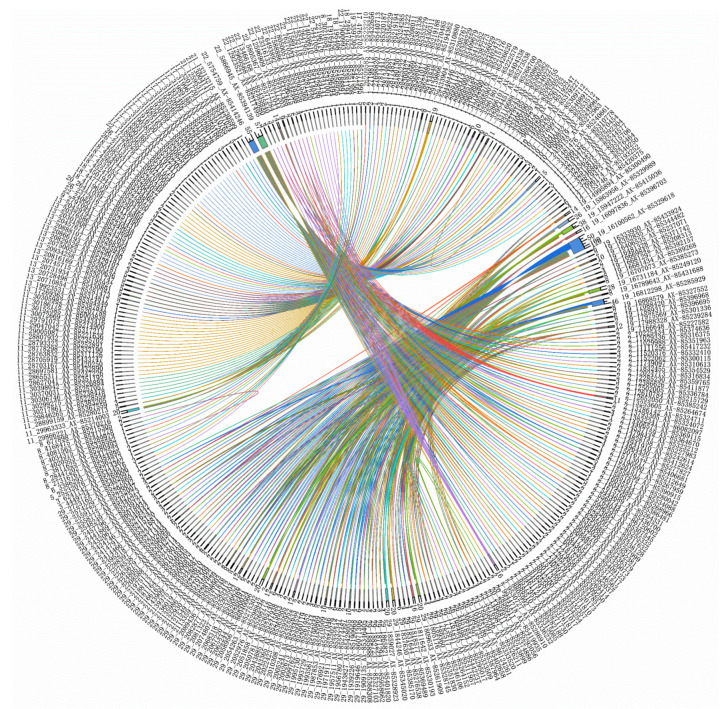
Chord diagram of additive-by-additive epistasis for the depth trait. The outer ring labels include chromosome numbers, base positions, and SNP IDs. The inner circle numbers represent the count of loci interacting with each SNP. (To avoid text overlap due to the extensive information, a smaller font size has been used. Please zoom in on the image for better clarity).

**Figure 5:**
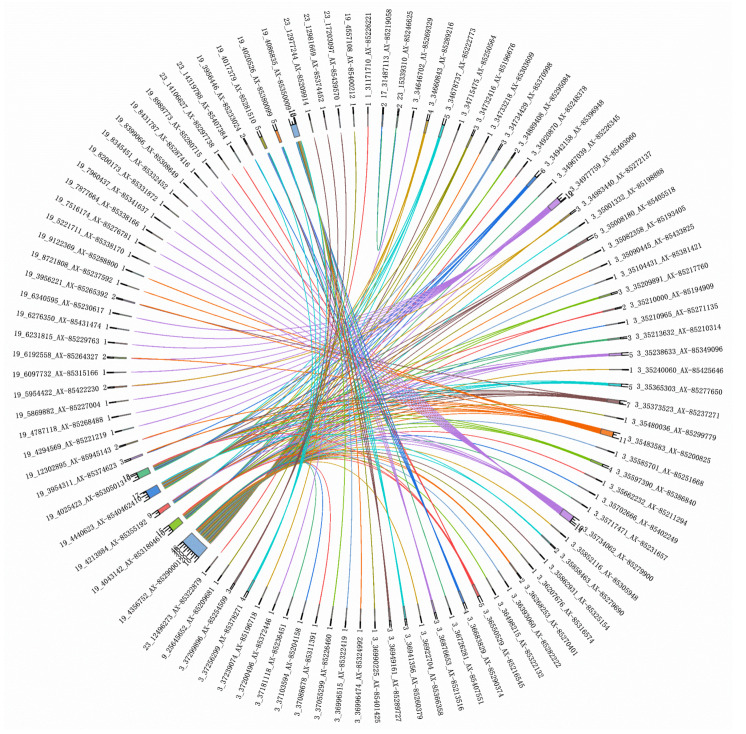
Chord diagram of additive-by-additive epistasis for the width trait. The outer ring labels include chromosome numbers, base positions, and SNP IDs. The inner circle numbers represent the count of loci interacting with each SNP.

**Figure 6:**
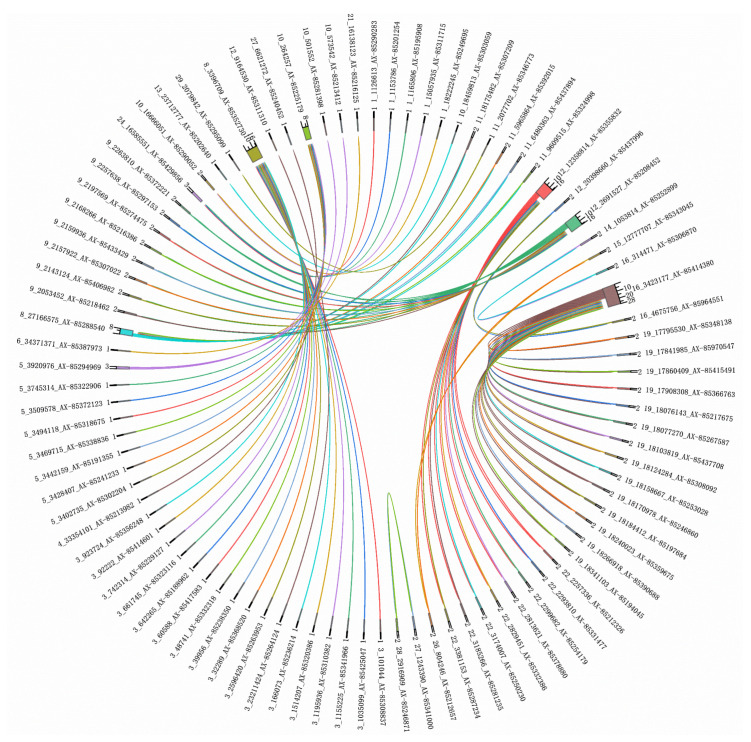
Chord diagram of additive-by-additive epistasis for the length trait. The outer ring labels include chromosome numbers, base positions, and SNP IDs. The inner circle numbers represent the count of loci interacting with each SNP.

We performed a genome-wide epistatic association analysis using the REMMAX function in the GMAT software and identified QTNs in additive-by-additive epistasis and additive-by-dominance epistasis (Figure 4, Figure 5 and Figure 6 and Table 4 and Table 5), while no QTNs were found in dominance-by-dominance epistasis or any other analyses. Detecting dominance-by-dominance epistasis is challenging due to its complexity, potential environmental influences, and limitations in statistical models. The absence of detected interactions in our study might be due to insufficient sample size or SNP density, the genetic basis of traits in catfish, environmental factors, and the constraints of the models used.

We only analyzed the candidate genes containing the QTN here. First, several candidate genes influence cranial or skeletal morphology. For example, forkhead box P2 (*foxp2*) is involved in speech and language disorders and plays a significant role in craniofacial development and bone remodeling [61,62,63,64]. It was found that upon stimulation of MC3T3-E1 osteoblastic cells with estradiol, there was a *rora*-mediated upregulation of bone morphogenetic protein 2 (BMP2) [65]. This may suggest that RAR-related orphan receptor A, paralog a (*roraa*), might interact with bone morphogenetic proteins (BMPs), thereby affecting cranial morphology. The paired box 5 (*pax5*) gene plays a critical role in controlling osteoclast development [66]. The *Pax* genes and *BMP* genes play a very important synergistic role in bone development, and there is an interaction between the *Pax* and *BMP* genes [67]. In studies on the facial morphology of European, Latin Americans, and Han Chinese, the *pax3* gene has been identified [68,69,70]. Therefore, we speculate that the *pax5* gene likely plays an important role in skull development as well. Potassium plays an important role in bones [71]. We identified the potassium voltage-gated channel subfamily A regulatory beta subunit 1a (*kcnab1a*) gene. According to the literature, potassium and bone morphogenetic proteins jointly influence bone development, and there are multiple interaction effects between them [72,73,74].

In addition to the genes influencing cranial or skeletal morphology, we also identified some other genes involved in bone development, which appear to participate in some form of interactive mechanism (Table 5). For example, the research report on the melatonin receptor 1A a (*mtnr1aa*) gene indicates that melatonin can inhibit the differentiation of osteoblasts and osteoclasts in zebrafish scales by blocking the Erk signaling pathway [75]. However, this contradicts the results obtained from in vitro models of mammalian cells and from in vivo studies where melatonin increased bone formation [76]. Additionally, it is known that the decrease in melatonin with age is associated with osteoporosis and bone loss [77], and melatonin treatment may help repair fractures by enhancing osteoblast generation [78]. Kruppel-like factor 3 (basic) (*klf3*) is considered an important regulatory factor in bone development [79]. It was found that inhibition of *klf3* might improve bone mass during osteogenesis. BMSC-derived exosomal miR-21-5p improved osteoporosis through regulating *klf3* [80]. Phosphoglucomutase 2 (*pgm2*) is found to be upregulated during osteoclast differentiation [81]. These genes all interact with the dedicator of cytokinesis 4b (*dock4b*) gene.

Research on the *dock4b* gene is limited. This gene is orthologous to the human *dock4* gene, which is expressed in bone marrow and plays a vital role in the development of precursor B cells into immature B cells [82]. It has been observed that *dock4* expression is reduced in bone marrow samples from patients with myelodysplastic syndromes (MDSs), highlighting its importance in normal bone marrow cell development [83]. Additionally, increased expression of *dock4* is associated with bone metastasis in early breast cancer patients, suggesting its potential involvement in tumor-related bone diseases [84]. Further studies integrating lncRNA and mRNA expression data have identified *dock4* as a potential biomarker for osteoporosis in the elderly. The *dock4* gene is regulated by two lncRNAs and four miRNAs [85], indicating its complex regulatory network. Based on medical and genetic research on the human *dock4* gene, we hypothesize that the *dock4b* gene also plays a significant role in skeletal development.

The *dock4b* gene appears in both additive-by-additive and additive-by-dominance epistasis analysis results and is located on chromosome 19. It interacts with several of the genes mentioned above, such as *mtnr1aa*, *klf3*, *pgm2*, and *pax5*, which all influence skeletal morphology and bone development and are located on chromosome 29. This suggests that there may be frequent genetic interaction mechanisms between chromosome 19 and chromosome 29 in bone development. Additionally, the specific role of *dock4b* in this network, particularly its function as a nodal point, requires further investigation.

**Figure 7:**
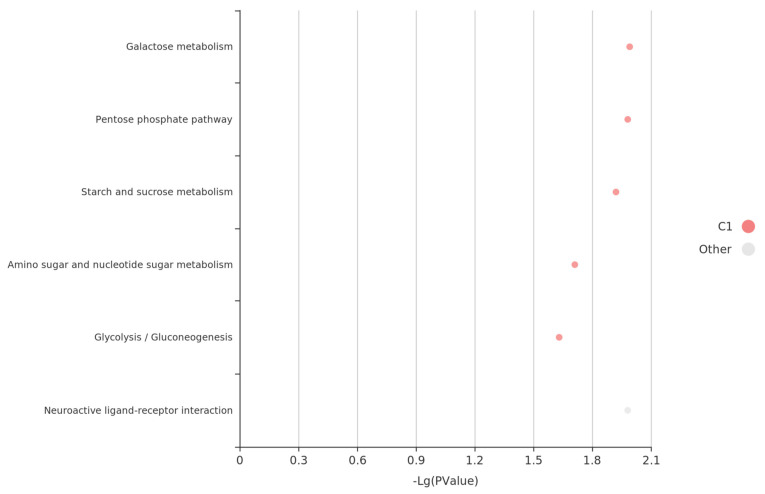
Pathway analysis of genes with epistatic interactions between chromosomes 19 and 29.

We conducted pathway analysis on candidate genes corresponding with the interaction loci between chromosomes 19 and 29, including *dock4b*, cyclin-dependent kinase 17 (*cdk17*), *foxp2*, *mtnr1aa*, *klf3*, *pgm2*, *pax5*, and glycine receptor, beta a (*glrba*). This analysis identified several key pathways (Figure 7) and led us to propose the following hypotheses: Metabolic regulation, encompassing carbohydrate, purine, and amino acid metabolism, plays a crucial role in head development by ensuring efficient energy metabolism and material supply. Additionally, neural and signaling pathways, particularly the neuroactive ligand–receptor pathway, may regulate head development by influencing head size traits through signaling mechanisms.

In addition to the genes mentioned above, the ryanodine receptor 3 (*RyR3*) gene is crucial for calcium signaling within muscle cells, which is essential for muscle contraction and overall skeletal muscle formation [86,87]. Gene–gene interaction analysis revealed that it interacts with the *kcnab1a* gene mentioned previously, which is responsible for potassium ion regulation.

## 4. Discussion

In this study, we applied novel analyses, including the GRAMMAR-Lambda and the GRAMMAR-Lambda-joint methods, to conduct a GWAS on head length, head width, and head depth traits in catfish. Our findings were compared with those of previous studies. Unlike the original study, where EMMAX failed to detect QTNs, our approach successfully identified multiple QTNs and candidate genes closely associated with skeletal development and morphology, including genes directly influencing skull structure.

Notably, the QFAM (conducted in PLINK) method that was used in the original study exhibited inflated test statistics in the QQ plot, leading to significant false positives and undermining the reliability of its QTN findings. In contrast, the GRAMMAR-Lambda method demonstrated ideal statistical properties, as evidenced by the QQ plots and genomic control values. This approach fully corrected the false negatives associated with the initial GRAMMAR approximation, effectively preserving the power of the test statistic and providing a robust framework for genomic control.

When comparing the candidate genes identified in our study with those reported in the original article, we found that only the *rab33a* gene appeared in both sets of results. The other candidate genes from the original article were absent in our findings. Given that the candidate genes in the original study were identified exclusively using the QFAM (conducted in PLINK) method—despite its significant false-positive issues—the credibility of those results is questionable. In contrast, our method identified several genes with direct relevance to craniofacial development, many of which were also highlighted in the literature review of the original study. This alignment further supports the reliability and robustness of our results.

Our GWAS analysis identified some genes directly impacting skeletal and skull morphology, such as *bmpr1bb*, *fgfrl1b*, and *nipbl*, which align closely with catfish head morphology traits. These genes have well-established roles in bone development; for instance, *bmpr1bb* is part of the Bone Morphogenetic Protein (BMP) receptor family, a key regulator of craniofacial morphology. In zebrafish, the absence of *fgfrl1b* results in craniofacial deformities, including suppressed cartilage formation in the branchial arches. The original study reviewed the influence of *bmp* and *fgfr* genes on skull morphology in its introduction and discussion, but these genes were not detected in their results, underscoring the reliability of our findings. Beyond genes that directly influence cranial development, we also identified *saraf* and *stim2a* genes, both part of the Store-Operated Calcium Entry (SOCE) mechanism. The SOCE mechanism directly affects skeletal cell function and tissue development by regulating intracellular calcium levels. Additionally, we detected multiple genes from the *SLC* and *Rab* gene families, both of which are critical to bone development and ion transport processes.

In our genome-wide epistatic association analysis, we identified the *foxp2* gene, which is known not only for its role in language disorders but also for its involvement in craniofacial development and bone remodeling. Furthermore, our analyses of additive-by-additive epistasis and additive-by-dominance epistasis highlighted *dock4b* on chromosome 19 as a significant factor, with interactions involving genes such as *mtnr1aa*, *klf3*, *pgm2*, and *pax5*, which are located on chromosome 29 and play roles in skeletal morphology and bone development. This suggests potential frequent interaction mechanisms between chromosomes 19 and 29 in bone development. Further studies are needed to explore the specific role of *dock4b* in this network, especially its function as a potential nodal point. The data used in this study were derived from SNP array genotyping. For an in-depth investigation into the complex interaction mechanisms between chromosomes 19 and 29, high-resolution genomic resequencing data may be required. Additionally, given that many quantitative traits are caused by minor-effect polygenes, obtaining more detailed genomic resequencing data would allow us to conduct a gene-based GWAS [88], enabling a more comprehensive analysis of head-related traits.

Many of the candidate genes we identified are supported by the extensive literature that confirms their roles in skeletal development. Some of these genes have homologs in humans, mice, and zebrafish that are also involved in bone development, while gene families such as *SLC* and *Rab* are known to impact skeletal growth. Other candidate genes are implicated in skeletal muscle development and neural processes or are expressed in bones, where they participate in hematopoiesis and immunity, potentially influencing bone growth indirectly.

Overall, our findings suggest that using multiple analytical methods in complex genetic analyses provides more comprehensive and accurate results, rather than relying on a few traditional methods. EMMAX and GEMMA failed to detect QTNs in this study, while our GRAMMAR-Lambda approach successfully identified some genes directly impacting skull morphology. This underscores the importance of not placing blind trust in established methods but instead exploring alternative approaches. We acknowledge the foundational work of the original researchers, and, while they may have been constrained by the analytical tools available at the time, we believe our findings complement and expand upon their research.

## 5. Conclusions

This study applied the GRAMMAR-Lambda method to investigate the genetic basis of head size traits in catfish, identifying significant quantitative trait nucleotides (QTNs) and candidate genes associated with cranial morphology. Compared with previous studies, our method provided more robust and accurate results, revealing key genes, such as *bmpr1bb*, *fgfrl1b*, and *nipbl*, that directly influence skeletal development. Additionally, we identified frequent genetic interactions between chromosomes 19 and 29, suggesting potential pathways that regulate head size. These discoveries contribute to our understanding of complex trait inheritance and offer practical implications for marker-assisted selection in aquaculture. Future studies using high-resolution genomic resequencing data may further elucidate the mechanisms involved, optimizing breeding strategies for economically valuable traits in fish.

In agricultural research, the ultimate goal of genetic mapping and analysis of trait inheritance mechanisms is their application to breeding programs. Genomic selection breeding requires accurate marker information, which necessitates minimizing background noise. Screening for true trait-related loci from a large pool of candidate genes is a labor-intensive and costly process, and false positives may indicate model limitations that can lead to the exclusion of true loci. As analytical methods improve, previously collected data can be reanalyzed to enhance resource efficiency and merit reevaluation. The ongoing discovery and validation of gene functions provide updated references for further analysis, making previous datasets valuable for reexamination and exploration. This approach not only conserves resources and maximizes the contributions of prior research but also helps to corroborate findings, forming a robust chain of evidence. Finally, we strongly recommend the GRAMMAR-Lambda method, developed by our team, for future GWAS analyses to enhance the reliability and depth of results.

## Figures and Tables

**Table 1:** Genomic control values for five different methods.

Method	Head Length	Head Width	Head Depth
GRAMMAR-Lambda	0.988	0.992	0.989
GRAMMAR-Lambda-joint	0.989	0.992	0.989
EMMAX	1.045	1.062	0.957
QFAM (conducted in PLINK)	1.199	1.312	0.934
GEMMA	1.028	1.022	0.959

**Table 2:** The QTNs associated with head size traits obtained from the GRAMMAR-Lambda method.

Trait	SNP ID	Chr	Position (bp)	Effect	Heritability (%)	−log10 (p)	Associated Gene (±150 kb)
length	AX-85293903	8	27,899,399	−0.068	1.775	7.974	*fgfrl1b* *slc7a11*
	AX-85344350	8	27,902,733	−0.042	1.527	6.937	*fgfrl1b* *slc7a11*
	AX-85223744	16	1,445,591	0.040	1.442	6.577	*bmpr1bb* *cplane1* *gdnfa* *nipblb* *nup155* *opn4xa* *pdlim5b* *slc1a3b*
	AX-85280970	4	33,281,317	−0.027	0.843	- *	*ankrd67* *ccnl1a* *pepd* *ptx3a*
	AX-85220227	23	18,795,869	0.020	0.624	- *	*lamtor2* *mex3a* *rab11al* *rab25b* *rpz4* *rpz5* *ubqln4*
width	AX-85285315	29	693,486	0.025	1.597	6.863	*chic2* *clta* *cplx2a* *fip1l1b* *gne* *mttp* *nansa* *rbpja* *saraf* *slc24a2* *slc34a2a* *spink4* *stim2a* *stra6l* *tdrd7a* *tmod1* *trnai-aau* *tspan5b* *xpa*
depth	AX-85396595	1	1,398,786	0.032	1.277	7.226	*irx2a* *irx4a* *nrsn1* *slc6a3*

* Only detected by the joint association analysis.

**Table 3:** QTNs found by a multi-trait GWAS using the GMAT software.

SNP ID	Chr	Position (bp)	Multivariate *p*-Value	Associated Gene (±150 kb)
AX-85401435	8	22,832,965	2.628 × 10^−12^	*ca5a* *rab33a* *slc7a5* *uba2*

**Table 4:** The QTNs with the greatest additive-by-additive epistasis according to the chord diagram.

Trait	SNP ID	Chr	Position (bp)	Gene Containing the SNP	Chromosomes Affected
depth	AX-85329618	19	16,100,562	*dock4b*	29
	AX-85285929	19	16,812,298	*cdk17*	29
	AX-85329989	19	15,863,956	*/*	29
	AX-85415036	19	15,947,222	*/*	29
	AX-85249120	19	16,731,184	*/*	29
	AX-85394139	22	5,866,945	*sra1*	2
	AX-85414246	22	5,754,759	*/*	2
width	AX-85290001	19	4,356,752	*grm8a*	3
	AX-85404624	19	4,440,623	*/*	3
	AX-85318046	19	4,043,142	*LOC108279457*	3
length	AX-85414380	16	3,423,177	*gramd1ba*	19
	AX-85352730	8	3,396,709	*/*	3
	AX-85355832	12	12,358,814	*zeb2b*	22
	AX-85208452	12	2,691,527	*LOC128634186*	9

**Table 5:** QTNs identified from additive-by-dominance epistasis analysis.

Trait	SNP ID	Chr	Position (bp)	Gene Containing the SNP	SNP ID	Chr	Position (bp)	Gene Containing the SNP	Effect	*p*-Value
depth	AX-85434516	5	25,232,076	*LOC108265909*	AX-85188891	18	195,408	*acsl6*	0.313035	1.83 × 10^−12^
	AX-85323180	11	1,008,327	/	AX-85342524	14	16,288,297	*roraa*	−0.22966	5.85 × 10^−13^
	AX-85323180	11	1,008,327	/	AX-85394793	14	16,321,107	/	−0.23027	4.23 × 10^−13^
	AX-85396703	19	16,097,836	*dock4b*	AX-85246106	29	1,782,517	/	−0.16464	1.23 × 10^−12^
	AX-85329618	19	16,100,562	*dock4b*	AX-85313565	29	1,802,187	*mtnr1aa*	−0.18043	1.71 × 10^−12^
	AX-85329618	19	16,100,562	*dock4b*	AX-85257177	29	1,897,225	*klf3*	−0.17712	1.71 × 10^−12^
	AX-85329618	19	16,100,562	*dock4b*	AX-85269499	29	1,910,208	/	−0.17793	1.54 × 10^−12^
	AX-85329618	19	16,100,562	*dock4b*	AX-85227046	29	1,976,921	*pgm2*	−0.18352	2.98 × 10^−13^
	AX-85329618	19	16,100,562	*dock4b*	AX-85324535	29	2,060,488	/	−0.18262	4.02 × 10^−13^
	AX-85329618	19	16,100,562	*dock4b*	AX-85320921	29	2,081,764	*pax5*	−0.18262	4.02 × 10^−13^
	AX-85329618	19	16,100,562	*dock4b*	AX-85325334	29	2,112,807	*pax5*	−0.18202	4.86 × 10^−13^
	AX-85329618	19	16,100,562	*dock4b*	AX-85336457	29	2,157,774	/	−0.17848	1.54 × 10^−12^
	AX-85329618	19	16,100,562	*dock4b*	AX-85215120	29	2,414,791	*glrba*	−0.18169	5.23 × 10^−13^
	AX-85433924	19	16,333,930	*foxp2*	AX-85246106	29	1,782,517	/	−0.17359	1.51 × 10^−12^
	AX-85237071	19	16,406,535	*foxp2*	AX-85246106	29	1,782,517	/	−0.18294	2.09 × 10^−14^
	AX-85367637	22	8,744,028	/	AX-85223510	11	2,136,759	/	0.15971	8.20 × 10^−13^
	AX-85371989	22	16,303,493	/	AX-85223510	11	2,136,759	/	0.197008	9.14 × 10^−13^
	AX-85371989	22	16,303,493	/	AX-85299031	11	2,213,073	*cntn4*	0.208702	1.84 × 10^−12^
	AX-85371989	22	16,303,493	/	AX-85422684	11	2,228,063	*cntn4*	0.208512	1.87 × 10^−12^
length	AX-85319825	3	31,181,550	/	AX-86070899	6	3,844,599	/	0.264966	1.59 × 10^−12^
	AX-85439845	3	31,199,287	/	AX-86070899	6	3,844,599	/	0.264978	1.51 × 10^−12^
width	AX-85250220	1	8,798,081	*efna3b*	AX-85406367	20	5,741,046	/	−0.1639	1.98 × 10^−12^
	AX-85340400	9	9,123,557	*RyR3*	AX-85311060	4	33,001,848	*kcnab1a*	−0.48076	2.04 × 10^−12^

## Data Availability

The dataset used in this study was downloaded from the public database figshare (https://figshare.com/s/7ad1c3f2a3d3cca9fcbe, accessed on 1 December 2024).

## Supplementary Materials

The following supporting information can be downloaded at: www.mdpi.com/article/10.3390/biology14010063/s1, Figure S1: Manhattan plots for head length. The plots in different colors in the front layer were generated from EMMAX (Efficient Mixed-Model Association eXpedited) and the plots in blue in the back layer were generated from QFAM (family-based association test for quantitative traits); Figure S2: Manhattan plots for head width. The plots in different colors in the front layer were generated from EMMAX (Efficient Mixed-Model Association eXpedited) and the plots in blue in the back layer were generated from QFAM (family-based association test for quantitative traits); Table S1: Information about regions associated with head length; Table S2: Information about regions associated with head width; Table S3: QTNs associated with head size traits obtained from the GRAMMAR-Lambda method; Table S4: QTNs found by a multi-trait GWAS using the GMAT software.
