# Supplementary material for: GRAMMAR-Lambda Delivers Efficient Understanding of the Genetic Basis for Head Size in Catfish
Source: Biology (Basel). 2025 Jan 13;14(1):63. doi: 10.3390/biology14010063 (PMC11760490; doi:10.3390/biology14010063)
Supplement: Supplementary file 1 [file biology-14-00063-s001.zip › biology-3389142-supplementary.docx]

**Figure S1:** Manhattan plots for head length. The plots in different colors in the front layer were generated from EMMAX (Efficient Mixed-Model Association eXpedited) and the plots in blue in the back layer were generated from QFAM (family-based association test for quantitative traits).

**Figure S2:** Manhattan plots for head width. The plots in different colors in the front layer were generated from EMMAX (Efficient Mixed-Model Association eXpedited) and the plots in blue in the back layer were generated from QFAM (family-based association test for quantitative traits).

**Table S1:** Information about regions associated with head length


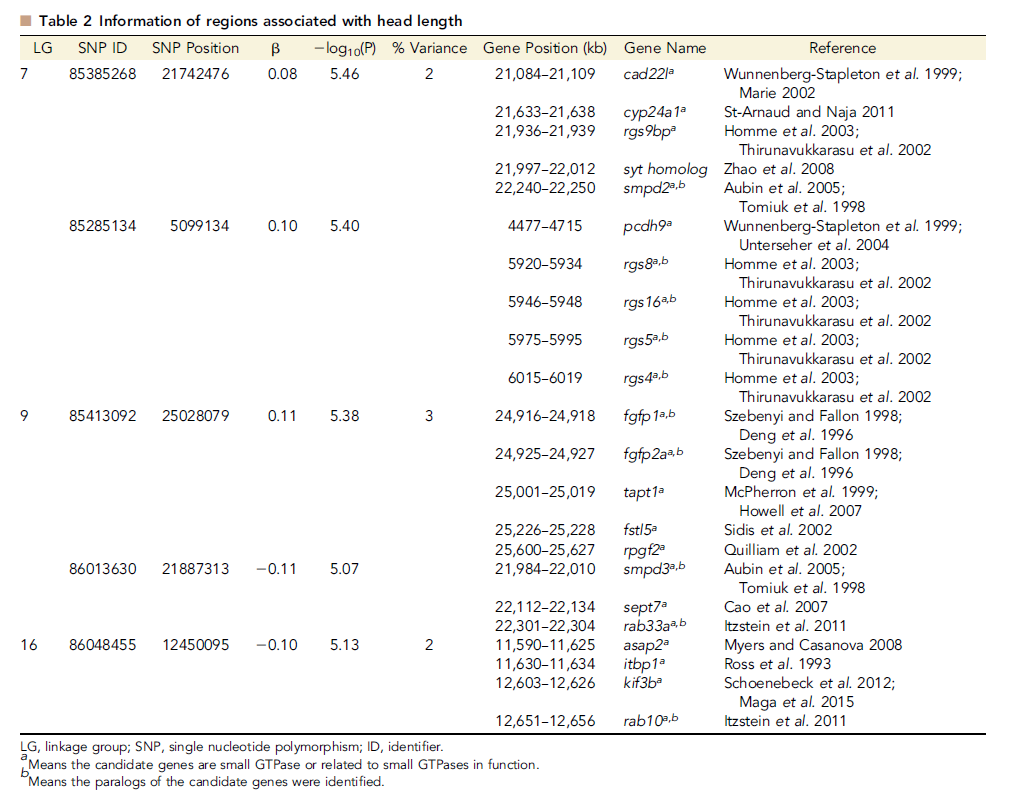


**Table S2:** Information about regions associated with head width


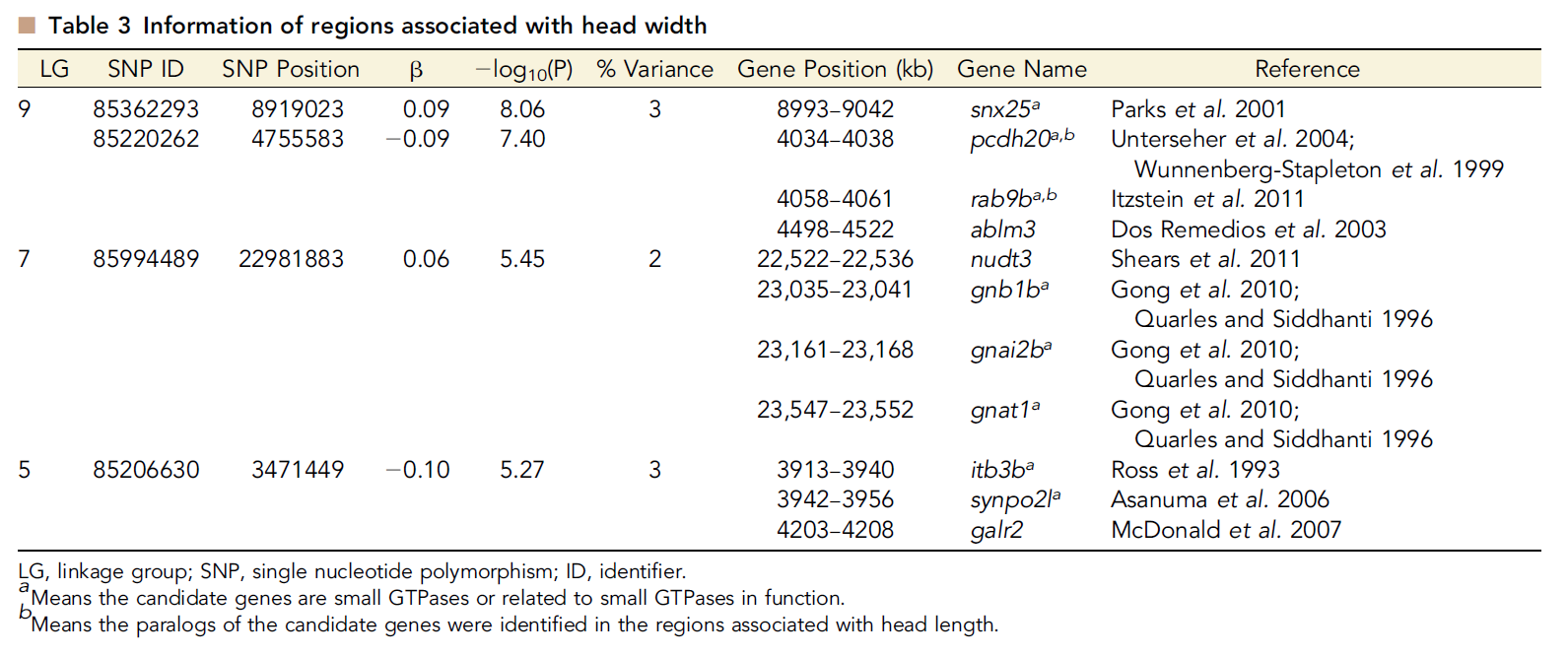


**Table S3** QTNs associated with head size traits obtained from the GRAMMAR-Lambda method.

| **Trait** | **SNP ID** | **Chr** | **Position (bp)** | **Effect** | **Heritability (%)** | **−log10(p)** | **Associated gene (±150kb)** |
| --- | --- | --- | --- | --- | --- | --- | --- |
| Length | AX-85293903 | 8 | 27899399 | -0.068 | 1.775 | 7.974 | fgfrl1b  slc7a11  LOC108268719  LOC108268722  LOC108268730  LOC108268744  LOC128628727  LOC128628787  LOC128633475 |
|  | AX-85344350 | 8 | 27902733 | -0.042 | 1.527 | 6.937 | fgfrl1b  slc7a11  LOC108268719  LOC108268722  LOC108268730  LOC108268744  LOC128628787  LOC128633475 |
|  | AX-85223744 | 16 | 1445591 | 0.040 | 1.442 | 6.577 | bmpr1bb  cplane1  gdnfa  nipblb  nup155  opn4xa  pdlim5b  slc1a3b  LOC108276963  LOC108276964  LOC108277018 |
|  | AX-85280970 | 4 | 33281317 | -0.027 | 0.843 | - * | ankrd67  ccnl1a  pepd  ptx3a  LOC108264140  LOC108264317  LOC108264318 |
|  | AX-85220227 | 23 | 18795869 | 0.020 | 0.624 | - * | lamtor2  mex3a  rab11al  rab25b  rpz4  rpz5  ubqln4  si:dkey-152b24.7  tmem176l.1  zmp:0000000881  LOC108256167  LOC108256173  LOC108256181  LOC108256183  LOC108256189  LOC108256190  LOC108256203  LOC124626227  LOC124626228 |
| width | AX-85285315 | 29 | 693486 | 0.025 | 1.597 | 6.863 | chic2  clta  cplx2a  fip1l1b  gne  mttp  nansa  rbpja  saraf  slc24a2  slc34a2a  spink4  stim2a  stra6l  tdrd7a  tmod1  trnai-aau  tspan5b  xpa  LOC108260686  LOC108260689  LOC108260691  LOC108260849  LOC108260901  LOC108260902  LOC124626524  LOC124626525  LOC128628759  LOC128629417 |
| depth | AX-85396595 | 1 | 1398786 | 0.032 | 1.277 | 7.226 | irx2a  irx4a  nrsn1  slc6a3  LOC108262776  LOC108263304  LOC108268542  LOC108272313  LOC108272386  LOC128635254  LOC128635265 |

* Only detected by the joint analysis

**Table S4** QTNs found by a multi-trait GWAS using the GMAT software

| **SNP ID** | **Chr** | **Position (bp)** | **Multivariate p-value** | **Associated gene (±150kb)** |
| --- | --- | --- | --- | --- |
| AX-85401435 | 8 | 22832965 | 2.628e-12 | ca5a  rab33a  slc7a5  uba2  si:ch211-159i8.4  LOC108268439  LOC108268543  LOC108269113  LOC108269114  LOC108269115 |
